# Targeting the NLRP3 Inflammasome-Related Pathways via Tianeptine Treatment-Suppressed Microglia Polarization to the M1 Phenotype in Lipopolysaccharide-Stimulated Cultures

**DOI:** 10.3390/ijms19071965

**Published:** 2018-07-05

**Authors:** Joanna Ślusarczyk, Ewa Trojan, Katarzyna Głombik, Anna Piotrowska, Bogusława Budziszewska, Marta Kubera, Katarzyna Popiołek-Barczyk, Władysław Lasoń, Joanna Mika, Agnieszka Basta-Kaim

**Affiliations:** 1 Department of Experimental Neuroendocrinology, Institute of Pharmacology, Polish Academy of Sciences, 12 Smetna St., 31-343 Krakow, Poland; slusarczyk_j@op.pl (J.Ś.); trojan@if-pan.krakow.pl (E.T.); glombik@if-pan.krakow.pl (K.G.); budzisz@if-pan.krakow.pl (B.B.); kubera@if-pan.krakow.pl (M.K.); lason@if-pan.krakow.pl (W.L.); 2 Department of Pharmacology of Pain, Institute of Pharmacology, Polish Academy of Sciences, 12 Smetna Str., 31-343 Krakow, Poland; annap@if-pan.krakow.pl (A.P.); popiolek@if-pan.krakow.pl (K.P.-B.); joamika@if-pan.krakow.pl (J.M.)

**Keywords:** tianeptine, M1/M2 microglia activation, cytokines, NLRP3 inflammasome, TLR4 intracellular pathways

## Abstract

An increasing body of evidence postulates that microglia are the main mediators of inflammation-related disorders, including depression. Since activated microglia produce a wide range of pro- and anti-inflammatory factors, the modulation of M1/M2 microglial polarization by antidepressants may be crucial in the treatment of depression. The current paper aimed to investigate the impact of tianeptine on the microglia’s viability/death parameters, and on M1/M2 microglial activation in response to lipopolysaccharide (LPS) stimulation. Furthermore, the molecular mechanisms via which tianeptine affected the LPS-evoked changes were investigated. The results revealed that tianeptine had partially protective effects on the changes in microglia viability/death evoked by LPS. Tianeptine attenuated microglia activation by decreasing the expression of cluster of differentiation 40 (CD40), and major histocompatibility complex class II (MHC II) markers, as well as the release of pro-inflammatory factors: interleukin (IL)-1β, IL-18, IL-6, tumor necrosis factor alpha (TNF-α), and chemokine CC motif ligand 2 (CCL2), and the production of nitric oxide and reactive oxygen species. In contrast, we did not observe an impact of tianeptine on M2 microglia measured by IL-4, IL-10, TGF-β, and insulin-like growth factor 1 (IGF-1) expression. Moreover, we demonstrated an inhibitory effect of tianeptine on the LPS-induced activation of the nucleotide-binding oligomerization domain-like (NOD-like) receptor pyrin-containing 3 inflammasome (NLRP3) inflammasome subunits, NLRP3 and caspase-1, as well as the ability of tianeptine to reduce Toll-like receptor 4 (TLR4) levels, as well as the phosphorylation of extracellular signal-related kinases 1 and 2 (ERK1/2) and of the nuclear factor kappa-light-chain-enhancer of activated B cells (NF-κB). Collectively, we demonstrated that tianeptine has protective properties and inhibits M1 polarization, thus attenuating the production of inflammatory mediators. Moreover, we found that M1 microglia suppression may be related to the NLRP3 inflammasome and TLR4 signaling. These findings suggest that a better understanding of the multifaceted mechanisms of tianeptine action on microglia may increase the effectiveness of therapy, where inflammation is a central hallmark.

## 1. Introduction

Numerous studies indicated that inflammatory processes and disturbed neuron-microglia interactions may play important roles in the pathogenesis of depression. Indeed, much evidence demonstrated that depression is associated with elevated levels of pro-inflammatory cytokines, mainly interleukin (IL)-1β and IL-18 [1,2,3], and with an increased rate of single nucleotide polymorphisms within pro-inflammatory genes [4]. Importantly, with brain inflammatory processes, a potentiation of neurodegenerative changes and diminished neurogenesis are observed in the course of depression. Furthermore, the inflammatory status may influence the function of the neuroendocrine system, the synaptic plasticity, and the metabolism of neurotransmitters, thereby stressing the pathological processes [5]. During inflammation, in addition to pro-inflammatory cytokines, other harmful mediators such as nitric oxide (NO) and reactive oxygen species (ROS) may also negatively affect brain neurogenesis, and participate in stress-induced depression [6].

An increasing body of evidence postulates that microglia, the resident immune cells in the central nervous system (CNS), can be recognized as the main mediators of inflammation-related disorders [7]. Interestingly, microglia cells manage the innate and adaptive immune responses not only in various pathological processes, but also during CNS repair [8,9]. Recently, an observation showed that active microglia can clear cellular debris via phagocytosis, thereby promoting tissue repair and regulating the response to pathogens. However, prolonged or excessive activation leads to a switch of microglia from regulatory to neurotoxic functions [8,9,10]. Microglia are commonly accepted as being the most sensitive indicators of brain condition [11,12].

Two well-established phenotypes of microglia include the M1 and M2 phenotypes, where the M1 phenotype is known to play a pro-inflammatory role, while the M2 phenotype, which is further subdivided into M2a, M2b, and M2c, is involved in anti-inflammatory/immunoregulatory processes [13,14]. Data indicated that activated M1 microglia change their morphology, and proliferate and enhance the expression of cluster-of-differentiation (CD) markers, including CD40 and major histocompatibility complex class II (MHC II) [15]. Moreover, microglia in the M1 state are a prominent source of pro-inflammatory cytokines such as IL-1β, IL-18, tumor necrosis factor alpha (TNF-α), and IL-6, as well as neurotoxic mediators such as NO, ROS, prostaglandin (PG) E2, and the superoxide anion [16,17]. In contrast, M2 polarized microglia have the ability to express anti-inflammatory cytokines, and are involved in the suppression of inflammation and in the restoration of homeostasis, as demonstrated by the release of transforming growth factor (TGF-β), insulin-like growth factor 1 (IGF-1), and IL-4 or IL-10 [14,18,19]. Alterations in microglia M1/M2 polarization are associated with excessive microglial inflammatory activation, and may play an important role in the development and progression of depressive disorders.

The nucleotide-binding oligomerization domain-like (NOD-like) receptor pyrin-containing 3 inflammasome (NLRP3) is highly expressed in microglia, and is important in the development of the neuroinflammation process [20]. NLRP3 is a multiprotein complex consisting of NLRP3, pro-caspase-1, and the apoptosis-associated speck-like protein containing a caspase recruitment domain (CARD; ASC). The NLRP3 complex is activated by various environmental and endogenous molecules, and is also indirectly activated by a primary component of the endotoxin from Gram-negative bacterial cell walls, lipopolysaccharide (LPS) [21]. Upon exposure to LPS, the transmembrane Toll-like receptor 4 (TLR4) is phosphorylated, consequently inducing intracellular signaling, resulting in the activation of mitogen-activated protein kinases (MAPKs; c-Jun N-terminal kinase (JNK), p38, and extracellular signal-related kinases (ERK1/2)), as well as nuclear factor kappa-light-chain-enhancer of activated B cells (NF-κB) signaling in the nucleus [22]. NF-κB promotes the transcription of NLRP3, as well as that of proIL-1β and proIL-18, which, after translocation, remain in the cytoplasm in their inactive forms. A second stimulus activates the NLRP3 inflammasome by facilitating the oligomerization of inactive NLRP3, ASC, and procaspase-1. This complex, in turn, catalyzes the conversion of procaspase-1 to caspase-1, which contributes to the production and secretion of M1 microglia mature pro-inflammatory cytokines, mainly IL-1β and IL-18.

So far, there are no data on whether or not NLRP3 inflammasome-related pathways may be a key target of various antidepressant drug actions. Among the antidepressants, tianeptine is an atypical drug with a mechanism of therapeutic action that is still undefined [23]. The neurochemical properties of tianeptine vary from other tricyclic and non-tricyclic antidepressants, because tianeptine was found to selectively potentiate serotonin uptake into rat brain synaptosomes [24]. Moreover, tianeptine has a wider profile of action, including effects on hippocampal neuroplasticity [25] and neuroprotection [26], as well as anticonvulsant [27] and antinociceptive efficacy [28]. Furthermore, tianeptine modulates hypothalamus-pituitary-adrenal (HPA) axis activity [24]. Importantly, this drug seems to have immunomodulatory properties, including the ability to attenuate the symptoms of sickness behavior induced by intraperitoneal (i.p.) injection of LPS or IL-1β, and to affect the central balance between pro- and anti-inflammatory cytokines (IL-1β/IL-10) [29]. In line with our previous data, we demonstrated that tianeptine has the ability to diminish depression-like behavior (in an animal model of depression) via normalization of the IGF-1 family network, as well as through cytokines and the chemokine/chemokine receptor axis [30,31]. Among others, chronic tianeptine treatment diminished malfunction in the fractalkine (CX3CL1) and fractalkine receptor (CX3CR1) axis. The unique localization of fractalkine, mainly on neurons, and CX3CR1 on microglia is responsible for correct neuron-microglia communication, as well as the resolution of inflammation. Based on the above-mentioned data, it is possible that the beneficial effect of tianeptine on the depressive-like behavioral changes may be related with tianeptine’s impact on microglia status. However, the polarization of microglia and the role of NLRP3 inflammasome pathways in response to tianeptine treatment remain uninvestigated.

Therefore, the experiments reported in this work were designed to study (1) the impact of tianeptine on microglial viability and death processes in basal and lipopolysaccharide-stimulated conditions; (2) the impact of tianeptine on microglia activation markers; (3) the possible tianeptine-induced modulation of the M1/M2 microglia phenotypes, estimated by the release of pro- and anti-inflammatory molecules, as well as that of reactive oxygen species and nitric oxide; and (4) the possible mechanism of the modulatory properties of tianeptine, including the intracellular signaling pathways associated with NLRP3.

## 2. Results

### 2.1. Tianeptine Modulates Changes in the Viability of LPS-Stimulated Microglial Cells

**Figure 1:**
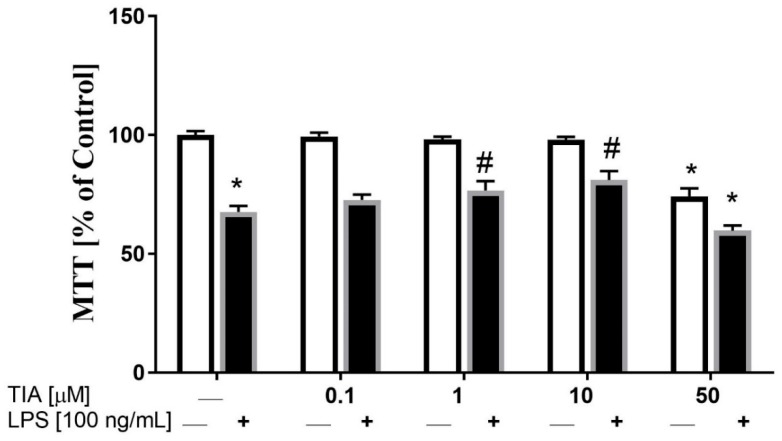
The influence of tianeptine (TIA) on cell viability (tetrazolium salt 3-(4,5-dimethylthiazol-2-yl)-2,5-diphenyltetrazolium bromide, MTT) in vehicle- and lipopolysaccharide (LPS)-treated primary microglial cells. Microglial cells were pretreated with various concentrations of tianeptine (TIA; 0.1, 1, 10, or 50 μM) for 30 min, and then with lipopolysaccharide (LPS; 100 ng/mL) for 24 h. The data are presented as the mean ± standard error of the mean (SEM) percentage of control (vehicle-treated cells) from independent experiments (*n* = 6 in each group). The results were statistically evaluated using two-way analysis of variance (ANOVA) with a Duncan post-hoc test to assess the differences between the treatment groups. Significant differences from the control group (vehicle-treated cells) are indicated by * *p* < 0.05; differences between LPS-treated groups and pre-treated with tianeptine and then stimulated with LPS groups are indicated by ^#^ *p* < 0.05.

In the first set of experiments, we examined the effect of different doses of tianeptine (TIA) on cell viability in unstimulated and LPS-stimulated microglia. As demonstrated in Figure 1, tianeptine only at 0.1, 1.0, and 10.0 μM did not evoke any significant changes in cell viability, as measured by the MTT reduction assay. However, exposure of control (unstimulated) microglial cells to tianeptine at 50 μM resulted in a significant decrease in viable cells (71% TIA vs. 100% control; *p* < 0.05). In contrast, we observed that 24 h of lipopolysaccharide (LPS, 100 ng/mL) stimulation significantly inhibited microglia viability, estimated by the MTT test (68% LPS vs. 100% control; *p* < 0.05; Figure 1). Interestingly, we showed that pretreatment with tianeptine (1 and 10 μM) attenuated the LPS-evoked decrease of microglia viability (79% TIA1 + LPS vs. 68% LPS, *p* < 0.05; 80% TIA10 + LPS vs. 68% LPS, *p* < 0.05), which is indicative of protective properties of tianeptine.

### 2.2. Tianeptine Modulates Changes in the Number of Propidium Iodide (PI)-Positive LPS-Stimulated Microglia Cells

**Figure 2:**
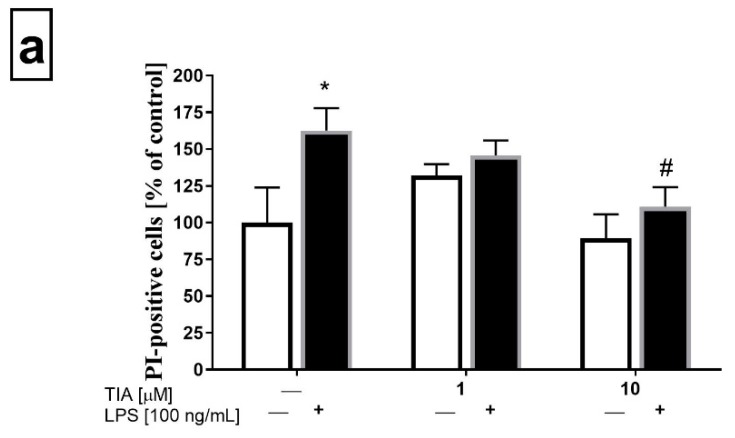

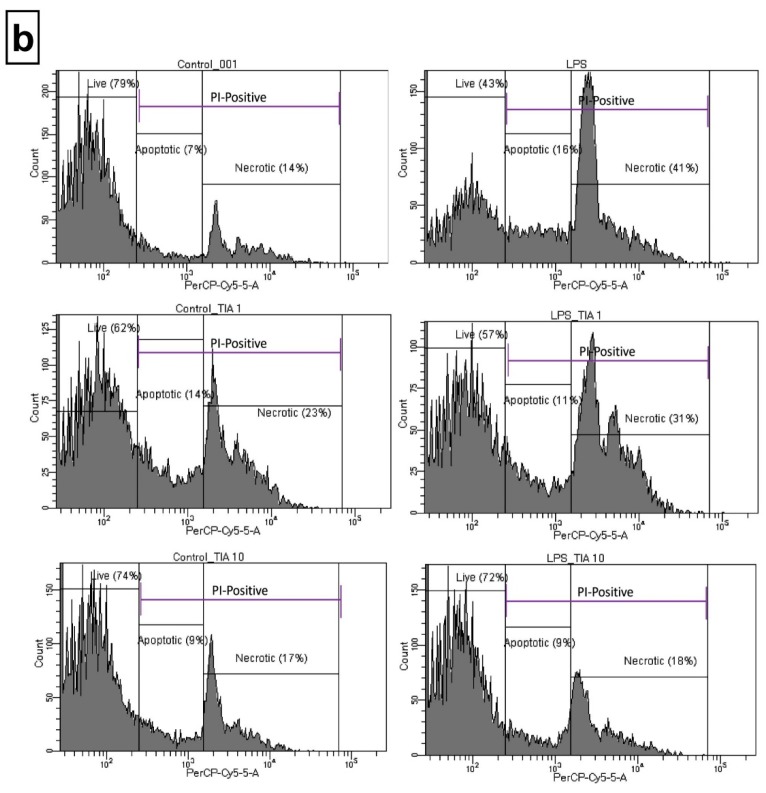
Effect of tianeptine (TIA) on the propidium iodide (PI)-positive cells measured using propidium iodide (PI) staining with flow cytometry analysis in vehicle- and LPS-treated primary microglial cells. (**a**) Illustration of the % of PI-positive cells from all samples (*n* = 7–8 in each group); (**b**) representative flow cytometric histograms. Microglial cells were pretreated with tianeptine (TIA; 1 or 10 μM) for 30 min, and then with lipopolysaccharide (LPS; 100 ng/mL) for 24 h. The data are presented as the mean ± SEM percentage of control (vehicle-treated cells) PI-positive cells, from independent experiments (*n* = 7–8 in each group). The results were statistically evaluated using two-way analysis of variance (ANOVA) with a Duncan post-hoc test to assess the differences between the treatment groups. Significant differences from the control group (vehicle-treated cells) are indicated by * *p* < 0.05; differences between LPS-treated groups and pre-treated with tianeptine and then stimulated with LPS groups are indicated by ^#^ *p* < 0.05.

To confirm the data obtained from the biochemical cell viability assay (MTT), flow cytometry analysis was performed to determine the propidium iodide (PI) staining in control (unstimulated) microglia cells, as well as in microglia cells 24 h after LPS stimulation. As demonstrated in Figure 2a, tianeptine only at 1.0 and 10 μM did not evoke statistically significant changes in cell viability as measured by PI uptake. As expected, LPS stimulation significantly enhanced the number of PI-positive microglia cells (162% LPS vs. 100% control, *p* < 0.05). Importantly, pretreatment with tianeptine (10 μM) significantly diminished the number of PI-positive microglia cells (110% TIA10 + LPS vs. 162% LPS, *p* < 0.05), as measured by flow cytometry analysis.

### 2.3. Tianeptine Diminishes NO Production, As Well As iNOS and ROS Levels in LPS-Stimulated Microglial Cells

**Figure 3:**
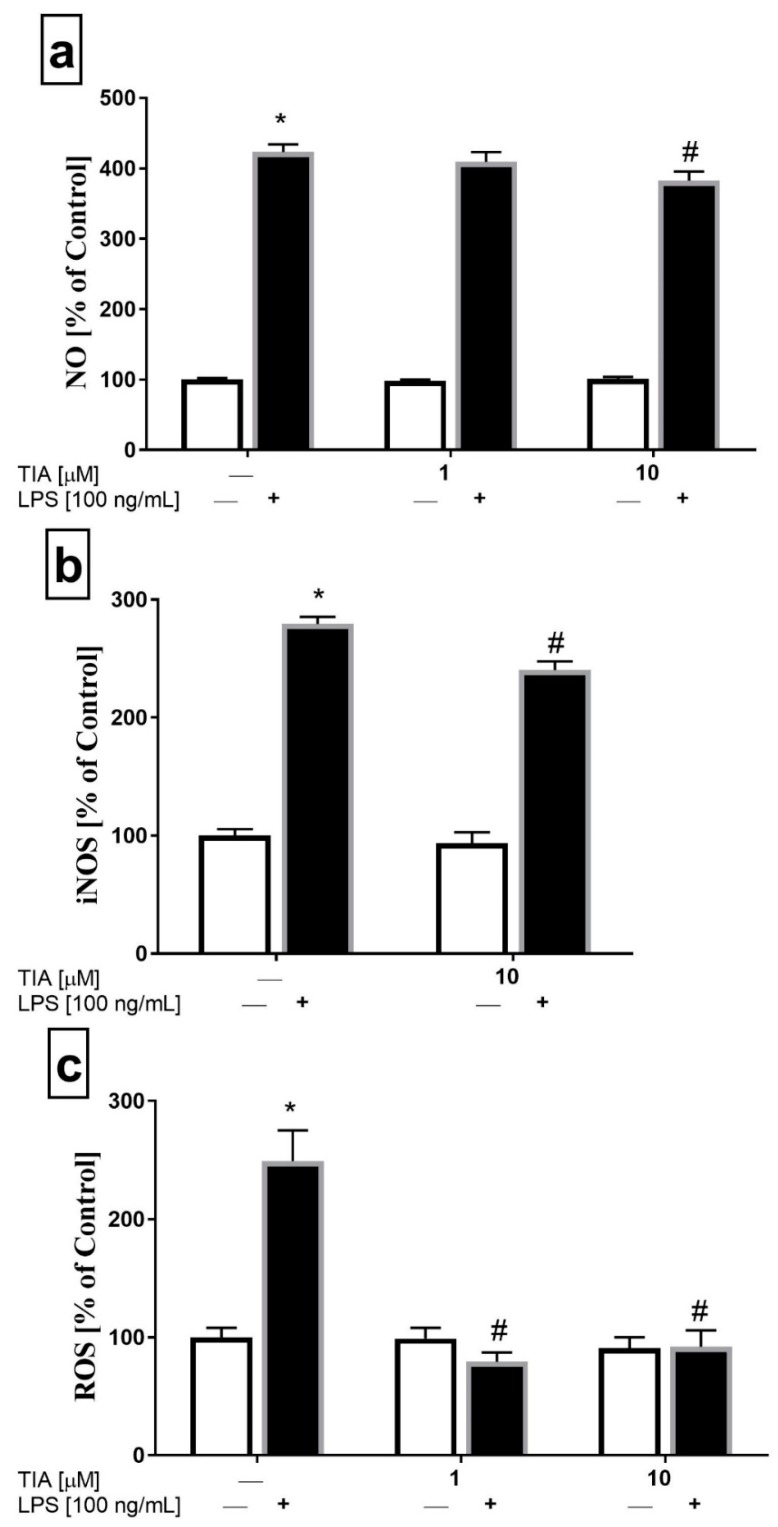
The effect of tianeptine (TIA) on nitric oxide (NO) release measured by the Griess reaction (**a**), the inducible nitric oxide synthase (iNOS) level (**b**) and the total intracellular reactive oxygen species (ROS) level (**c**) in vehicle- and LPS-treated primary microglial cells. Microglial cells were pretreated with tianeptine (TIA; 1 or 10 μM) for 30 min, and then with lipopolysaccharide (LPS; 100 ng/mL) for 24 h. The data are presented as the mean ± SEM percentage of control (vehicle-treated cells) from independent experiments (*n* = 6 in each group). The results were statistically evaluated using two-way analysis of variance (ANOVA) with a Duncan post-hoc test to assess the differences between the treatment groups. Significant differences from the control group (vehicle-treated cells) are indicated by * *p* < 0.05; differences between LPS-treated groups and pre-treated with tianeptine and then stimulated with LPS groups are indicated by ^#^ *p* < 0.05.

To assess the impact of tianeptine on NO secretion in microglia, we used an assay based on the Griess reaction. Microglia cells were stimulated for 24 h, and then NO release was measured. As shown in Figure 3a, microglia cells stimulated by LPS showed enhanced NO production (414% LPS vs. 100% control, *p* < 0.05). Moreover, tianeptine pretreatment for 30 min, but only at the dose of 10 μM, slightly reduced the LPS-evoked increase in NO production (365% TIA10 + LPS vs. 414% LPS, *p* < 0.05; Figure 3a). Consistent with the downregulation of NO production, we demonstrated using the ELISA method that tianeptine (10 μM) attenuated LPS-induced iNOS protein level increase (240% TIA10 + LPS vs. 279% LPS, *p* < 0.05; Figure 3b). These results indicate that tianeptine may attenuate NO production via the inhibition iNOS expression in LPS-stimulated microglia.

Next, we measured the total intracellular reactive oxygen species (ROS) production. The ROS level was tested in both unstimulated microglia cells and microglia cells stimulated with LPS for 24 h. As expected, LPS stimulation significantly increased ROS formation (248% LPS vs. 100% control, *p* < 0.05). Interestingly, tianeptine pretreatment for 30 min at 1 μM and 10 μM effectively decreased the LPS-evoked increase in ROS production (79% TIA1 + LPS vs. 248% LPS, *p* < 0.05; 91% TIA10 + LPS vs. 248% LPS, *p* < 0.05; Figure 3c). Based on the all findings described above, the concentration of 10 μM was selected for further testing.

### 2.4. Tianeptine Reduces the Expression of Microglial Activation Markers in LPS-Stimulated Cells

Microglial activation was previously reported to be associated with marked increases in CD40 and MHC II expression [32,33]. In our study, 24 h of microglial cell stimulation with LPS caused a significant increase in both CD40 and MHC II messenger RNA (mRNA) expression (*p* < 0.05). Interestingly, pretreatment of cells with tianeptine at the dose of 10 μM effectively inhibited the upregulation of CD40 and MHC II (*p* < 0.05; Table 1). These observations show that tianeptine has a significant inhibitory effect on LPS-evoked microglial cell activation.

### 2.5. Tianeptine Suppresses Microglia Polarization to the M1 but Not the M2 Phenotype in LPS-Stimulated Cells

To evaluate the impact of tianeptine on the expression of pro-inflammatory factors, IL-1β, IL-18, TNF-α, IL-6, and CCL2, as well as anti-inflammatory factors, IL-4, IL-10, TGF-β and IGF-1, in LPS-stimulated microglia cells, we measured both the gene and protein expressions of the above-mentioned factors. In control (unstimulated) cells, tianeptine (10 μM) did not affect the mRNA expression of the tested pro-inflammatory or anti-inflammatory factors, with the exception of TGF-β mRNA suppression (Table 1). As expected, the stimulation of primary microglial cells for 6 h with LPS led to a significant enhancement in the mRNA expression of M1 microglia markers, IL-1β, IL-18, TNF-α, IL-6, and chemokine CCL2 (*p* < 0.05). Moreover, we observed that 24-h LPS treatment affected the expression of M2 markers, significantly enhancing IL-10 expression (*p* < 0.05), while strongly diminishing TGF-β (*p* < 0.05) and IGF-1 (*p* < 0.05) mRNA expression.

Interestingly, pretreatment with tianeptine at 10 μM suppressed the LPS-evoked overexpression of all pro-inflammatory factors (*p* < 0.05). However, tianeptine did not significantly modulate the changes in anti-inflammatory gene expression evoked by LPS (Table 1).

**Figure 4:**
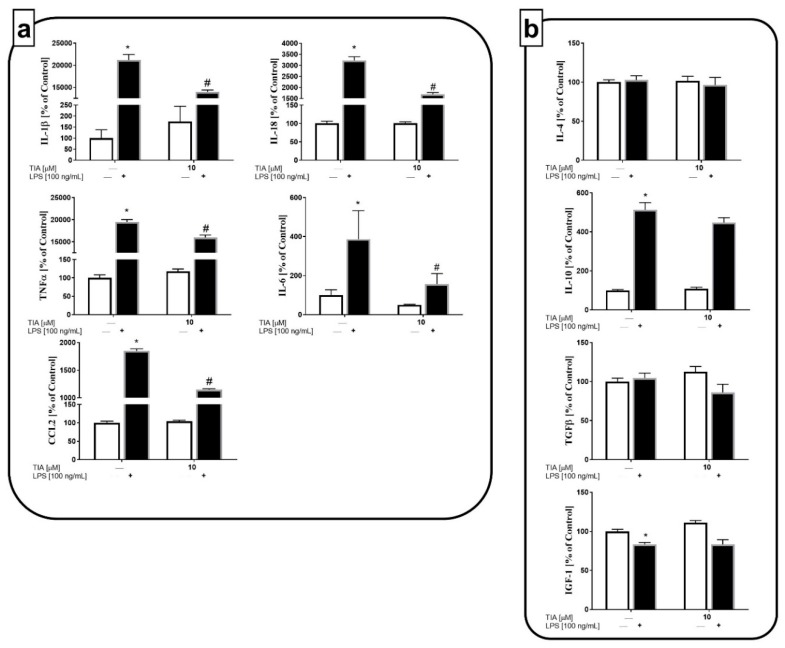
The effect of tianeptine (TIA) on the levels of pro-inflammatory (interleukin (IL)-1β, IL-18, tumor necrosis factor alpha (TNF-α), IL-6, and chemokine CC motif ligand 2 (CCL2)) (**a**) and anti-inflammatory (IL-4, IL-10, transforming growth factor (TGF-β), and insulin-like growth factor 1 (IGF-1)) (**b**) factors in vehicle- and LPS-treated primary microglial cells. Microglial cells were pretreated with tianeptine (TIA; 10 μM) for 30 min, and then with lipopolysaccharide (LPS; 100 ng/mL) for 24 h. The data are presented as the mean ± SEM percentage of control (vehicle-treated cells) from independent experiments (*n* = 6 in each group). The results were statistically evaluated using two-way analysis of variance (ANOVA) with a Duncan post-hoc test to assess the differences between the treatment groups. Significant differences from the control group (vehicle-treated cells) are indicated by * *p* < 0.05; differences between LPS-treated groups and pre-treated with tianeptine and then stimulated with LPS groups are indicated by ^#^ *p* < 0.05.

In addition to measuring the mRNA expression of pro- and anti-inflammatory factors, we examined the impact of tianeptine on all mentioned protein levels. In line with the results described above, we observed that 24-h LPS treatment dramatically enhanced the production of all pro-inflammatory cytokines, IL-1β (21,172% LPS vs. 100% control, *p* < 0.05), IL-18 (3224% LPS vs. 100% control, *p* < 0.05), TNF-α (19,465% LPS vs. 100% control, *p* < 0.05), IL-6 (380% LPS vs. 100% control, *p* < 0.05), and chemokine CCL2 (1847% LPS vs. 100% control, *p* < 0.05; Figure 4a). Importantly pretreatment with tianeptine significantly downregulated the LPS-enhanced levels of all pro-inflammatory factors examined, IL-1β (13,948% TIA10 + LPS vs. 21,172% LPS, *p* < 0.05), IL-18 (1688% TIA10 + LPS vs. 3224% LPS, *p* < 0.05), TNF-α (15,987% TIA10 + LPS vs. 19,465% LPS, *p* < 0.05), IL-6 (120% TIA10 + LPS vs. 380% LPS, *p* < 0.05), and CCL2 (1147% TIA10 + LPS vs. 1847% LPS, *p* < 0.05; Figure 4a). Moreover, we demonstrated that 24 h of stimulation by LPS did not affect TGF-β or IL-4 production, while enhancing IL-10 (513% LPS vs. 100% control, *p* < 0.05) release, and diminishing the production of IGF-1 (83% LPS vs. 100% control, *p* < 0.05) by microglia cells (Figure 4b). These observations demonstrate that the anti-inflammatory action of tianeptine is preferentially related to its suppressive effect on the microglia M1 phenotype.

### 2.6. Tianeptine Reduces the Level of TLR4 in LPS-Activated Microglia Cells

**Figure 5:**
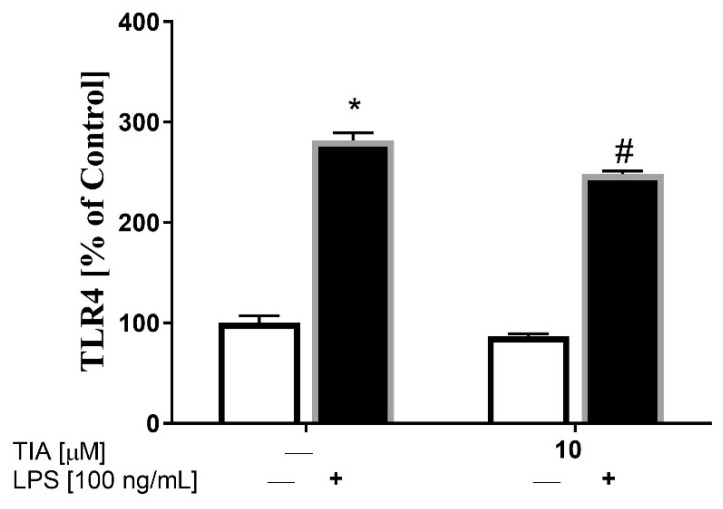
The effect of tianeptine (TIA) on Toll-like receptor 4 (TLR4) levels measured using an ELISA kit in vehicle- and LPS-treated primary microglial cells. Microglial cells were pretreated with tianeptine (TIA; 10 μM) for 30 min, and then with lipopolysaccharide (LPS; 100 ng/mL) for 24 h. The data are presented as the mean ± SEM percentage of control (vehicle-treated cells) from independent experiments (*n* = 6 in each group). The results were statistically evaluated using two-way analysis of variance (ANOVA) with a Duncan post-hoc test to assess the differences between the treatment groups. Significant differences from the control group (vehicle-treated cells) are indicated by * *p* < 0.05; differences between LPS-treated groups and pre-treated with tianeptine and then stimulated with LPS groups are indicated by ^#^ *p* < 0.05.

TLR4 is known to be a major LPS signaling receptor that leads to the activation of intracellular MAPK pathways, such as ERK1/2, JNK, and p38, as well as of transcription factors, including NF-κB, which, in turn, enhance the synthesis of inflammatory genes. Therefore, we firstly examined whether tianeptine may affect LPS-induced microglia activation on the TLR4 receptor level. To study the molecular mechanism of tianeptine action, we focused only on the dose of 10 μM, because it was the most effective in the experiments described above. We showed using the ELISA method that, in control (unstimulated) cells, tianeptine did not affect TLR4 protein expression in microglia. However, as shown in Figure 5, 24 h of LPS stimulation upregulated TLR4 protein level (281% LPS vs. 100% control, *p* < 0.05), while pretreatment with tianeptine for 30 min reduced the LPS-induced enhancement of TLR4 protein expression (248% TIA10 + LPS vs. 281% LPS, *p* < 0.05).

### 2.7. Tianeptine Inhibits LPS-Induced Microglial Activation through the ERK1/2 and NF-κB Pathways

**Figure 6:**
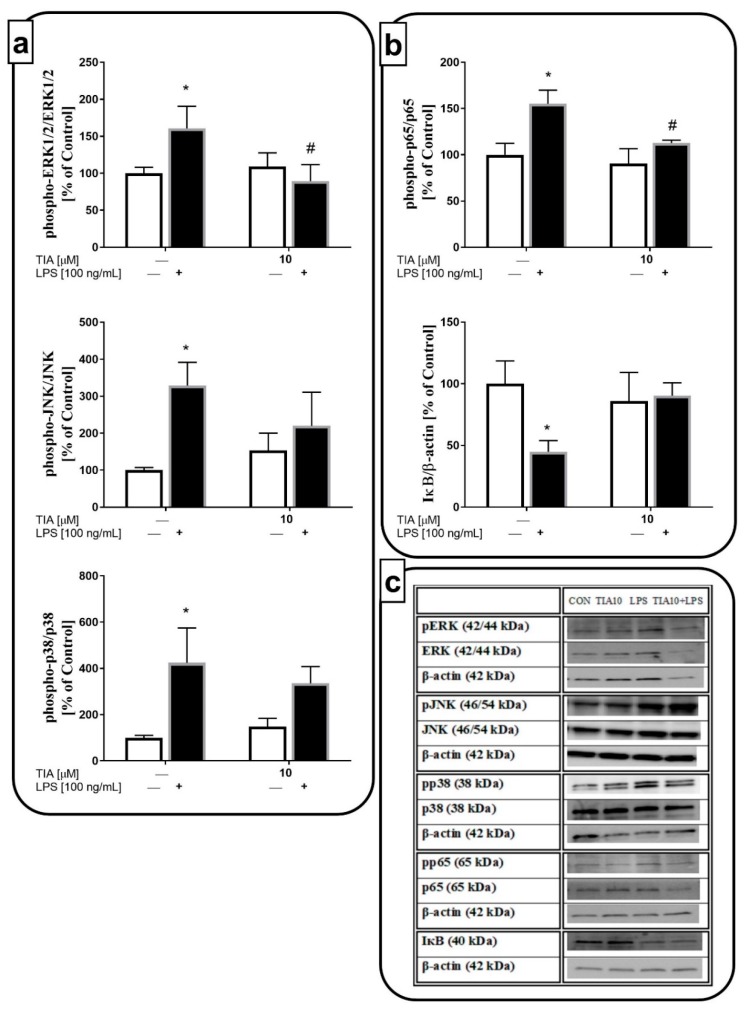
The effect of tianeptine (TIA) on (**a**) mitogen-activated protein kinases (MAPK) and (**b**) nuclear factor kappa-light-chain-enhancer of activated B cells (NF-κB) pathways measured using western blot analyses in vehicle- and LPS-treated primary microglial cells; (**c**) representative immunoblots. Microglial cells were pretreated with tianeptine (TIA; 10 μM) for 30 min, and then with lipopolysaccharide (LPS; 100 ng/mL) for 30 min. The data were evaluated as a ratio of the phosphorylated form of each kinase/total amount of kinase and presented as the mean ± SEM percentage of control (vehicle-treated cells) from independent experiments (*n* = 6 in each group). The results were statistically evaluated using two-way analysis of variance (ANOVA) with a Duncan post-hoc test to assess the differences between the treatment groups. Significant differences from the control group (vehicle-treated cells) are indicated by * *p* < 0.05; differences between LPS-treated groups and pre-treated with tianeptine and then stimulated with LPS groups are indicated by ^#^ *p* < 0.05 (see Supplementary materials).

To further investigate the intracellular mechanism of the anti-inflammatory effects of LPS-evoked M1 microglial activation, we used the dose of 10 μM tianeptine. We demonstrated that LPS stimulation for 30 min led to activation of the kinases, ERK1/2 (160% LPS vs. 100% control, *p* < 0.05), JNK (328% LPS vs. 100% control, *p* < 0.05), and p38 (425% LPS vs. 100% control, *p* < 0.05). Pretreatment with tianeptine markedly blocked the LPS-evoked ERK1/2 phosphorylation (89% TIA10 + LPS vs. 160% LPS, *p* < 0.05). On the other hand, tianeptine showed no effect on the LPS-evoked increase in phosphorylation of the other MAPKs, JNK and p-38 (Figure 6a). Next, we measured the phosphorylation level of the p65 NF-κB subunit and the IκB protein, an inhibitor of the NF-κB complex. As shown in Figure 6b, LPS stimulation enhanced the phosphorylation of the p65 subunit (155% LPS vs. 100% control, *p* < 0.05), and diminished the level of IκB (44% LPS vs. 100% control, *p* < 0.05). Tianeptine pretreatment significantly suppressed NF-κB phosphorylation (112% TIA10 + LPS vs. 155% LPS, *p* < 0.05). Therefore, the ERK1/2 and NF-κB pathways may be postulated as important in the anti-inflammatory action of tianeptine in microglial cultures stimulated by LPS.

### 2.8. Tianeptine Diminishes NLRP3 Inflammasome Activation in LPS-Stimulated Microglial Cells

**Figure 7:**
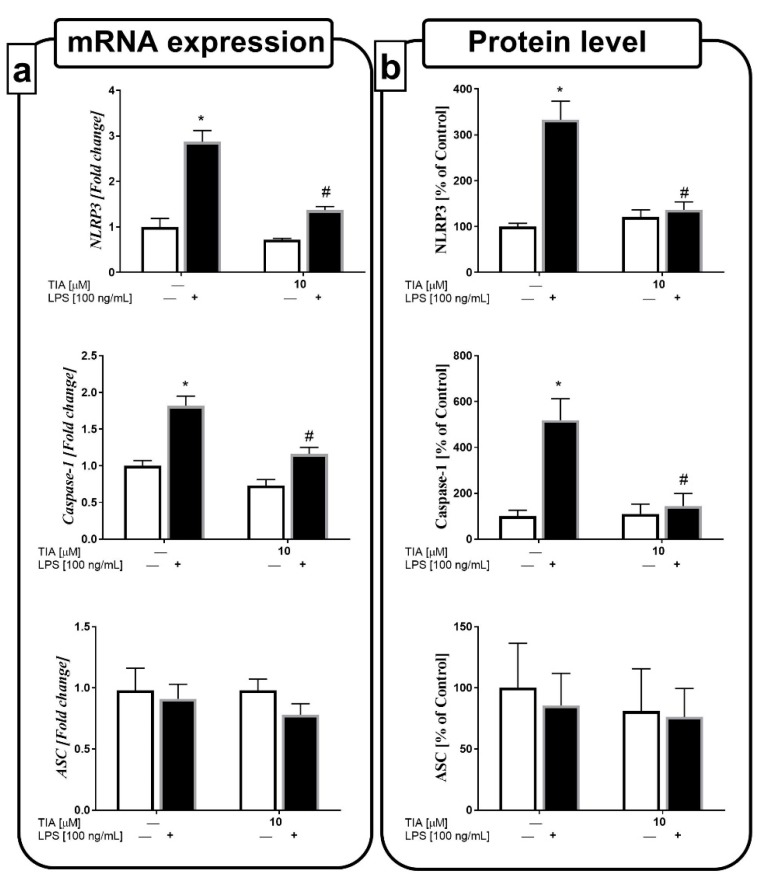
The effect of tianeptine (TIA) on the messenger RNA (mRNA) (**a**) and protein expressions (**b**) of all nucleotide-binding oligomerization domain-like (NOD-like) receptor pyrin-containing 3 inflammasome (NLRP3) subunits, measured in vehicle- and LPS-treated primary microglial cells. Microglial cells were pretreated with tianeptine (TIA; 10 μM) for 30 min, and then with lipopolysaccharide (LPS; 100 ng/mL) for 24 h. The qRT-PCR data are presented as the average fold ± SEM from independent experiments (*n* = 6 in each group). Protein levels obtained from the ELISA test are presented as the mean ± SEM percentage of control (vehicle-treated cells) from independent experiments (*n* = 6 in each group). The results were statistically evaluated using two-way analysis of variance (ANOVA) with a Duncan post-hoc test to assess the differences between the treatment groups. Significant differences from the control group (vehicle-treated cells) are indicated by * *p* < 0.05; differences between LPS-treated groups and pre-treated with tianeptine and then stimulated with LPS groups are indicated by ^#^ *p* < 0.05.

Recent studies indicated that the NLRP3 inflammasome is an important factor involved in the secretion of pro-inflammatory cytokines, mainly IL-1β and IL-18. Therefore, we estimated the impact of tianeptine on both the gene expression and protein levels of components of the NLRP3 inflammasome, such as NLRP3, caspase-1, and ASC. We observed that 24 h of LPS stimulation upregulated the gene expressions of NLRP3 and caspase-1, as well as the levels of both proteins (333% LPS vs. 100% control, *p* < 0.05; 518% LPS vs. 100% control, *p* < 0.05). Furthermore, we demonstrated, for the first time, that tianeptine pretreatment for 30 min at 10 μM effectively decreased the LPS-evoked upregulation in the protein levels of the NLRP3 and caspase-1 inflammasome subunits (136% TIA10 + LPS vs. 333% LPS, *p* < 0.05; 143% TIA10 + LPS vs. 518% LPS, *p* < 0.05; Figure 7a,b).

## 3. Discussion

The data from this study showed that tianeptine, an atypical antidepressant drug, suppresses microglia M1 polarization in response to lipopolysaccharide stimulation. Interestingly, the molecular mechanisms underlying the beneficial, anti-inflammatory effect of tianeptine are based on the inhibition of NLRP3 inflammasome activation and Toll-like receptor 4-related pathways. Tianeptine did not have an impact on “alternative” M2 microglia polarization.

In the presented study, bacterial endotoxin LPS administration was chosen as an experimental model to induce microglial activation. Although this model has some limitations, as LPS treatment does not totally reflect the neuroinflammation observed in pathological conditions (such as depression), it is commonly accepted for the evaluation of the impact of antidepressants on microglial activity [34,35,36]. Microglia are myeloid cells, the most important component of the brain’s immune system. As mononuclear phagocytes in the brain, microglia play a crucial role in maintaining normal brain functions in both physiological and pathological conditions [37]. The morphology of microglia reveals the different activation states of these cells. In a healthy brain, microglia have a ramified shape with extensively branched processes that contact neurons, astrocytes, and blood vessels, and continually survey and monitor changes in the local milieu [38]. On the other hand, once activated by stress, a bacterial endotoxin, or pro-inflammatory stimuli, microglia develop an ameboid morphology, characterized by cell-body enlargement and the presence of numerous cytoplasmic vacuoles, which are associated with phagocytosis and pro-inflammatory functions [15,39]. In addition to the morphological changes in response to LPS, microglia upregulate a number of surface proteins (MHC II, CD40, and CD68), and show enhanced release of cytokines (IL-18, IL-1β, TNF-α, and IL-6), chemokines (CCL2), and other neurotoxic mediators, such as NO and prostaglandin [40,41].

At the beginning of our study, we demonstrated that tianeptine attenuated the LPS-evoked decrease in microglia viability (MTT test). However, tianeptine showed slightly weaker (only at a dose of 10 μM) protective effects measured by PI uptake in flow cytometry. The discrepancies observed in the tianeptine action in our study may be due to the tests that we used to measure different aspects of the vital status of microglia cells. Generally, MTT yellow dye is converted to insoluble purple precipitates by mitochondria in live cells, thereby illustrating the metabolic activity of the cells [42]. On the other hand, the PI assay is used as a DNA stain to evaluate cell viability, since it cannot cross the membrane of live cells, making it useful in the differentiation of necrotic, apoptotic, and viable cells.

The first main findings of our study were that tianeptine not only decreased the LPS-evoked upregulation of MHC II and CD40, but also inhibited the LPS-evoked change in gene expression and the release of IL-1β, IL-18, TNF-α, and IL-6 by microglial cells. Furthermore, this drug effectively suppressed the production of CCL2, which plays a key role in the regulation of inflammatory processes [43]. Based on the available data, our observations show the suppressive potential of tianeptine on M1 polarization. Previously, chronic treatment with tianeptine was reported to attenuate the pro-inflammatory effects of IL-1β in some brain areas in mice [44]. Moreover, tianeptine was found to antagonize the behavioral effects of LPS [45], and inhibit LPS-induced c-fos expression in the rat paraventricular nucleus [46]. In contrast, tianeptine did not modulate the release of IL-4, TGF-β, IL-10, or neurotrophic factor IGF-1. The lack of an impact of tianeptine on the release of these beneficial factors may be due to the experimental schema, which was limited to the determination of the levels of the mentioned factors only after 24 h of stimulation. On the other hand, this lack of an effect may suggest that, although tianeptine exerts a strong anti-inflammatory effect and modifies the M1/M2 balance, it does not participate in the resolution of inflammation via M2 microglia activation.

The anti-inflammatory properties of tianeptine in our study were also confirmed by its partial inhibitory effects on nitric oxide production and iNOS level. The excessive production of NO by activated microglia leads to the formation of peroxynitrite via a reaction with superoxide, which kills cells by disturbing mitochondrial processes [47]. Data suggested that NO may be involved in the pathogenesis of depression due to its ability to modify monoaminergic transmission [48]. Indeed, studies showed increased levels of NO in patients suffering from depression compared to those in control subjects [49]; however, the exact role of NO in the pathogenesis of depression remains controversial because of the ability of these factors to promote both neuronal survival and neuronal death, depending on their localization and concentration [50]. Apart from nitric oxide, microglia activation also promotes the release of ROS, which include superoxide (O_2_^−^), hydrogen peroxide (H_2_O_2_), and the hydroxyl radical (●OH) [51,52]. In physiological conditions, ROS maintain cell homeostasis, while in pathological conditions, increased ROS levels elevate the expression of apoptotic genes and inflammatory mediators, resulting in inflammation, cell death, and disease progression [53]. Moreover, cytokines such as TNF-α, IL-1β, and IFN-γ act in a feedback loop to stimulate ROS production via the activation of nicotinamide adenine dinucleotide phosphate (NADPH) oxidase, resulting in redox disequilibrium and oxidative damage [54,55]. The presented results clearly show that tianeptine inhibited the LPS-stimulated production of ROS in microglia cells; therefore, the beneficial influence of tianeptine on ROS secretion may be suggested to be the result of both a rebalance of the redox equilibrium and a downregulation of elevated cytokine production in activated microglia.

The second aim of our research focused on the evaluation of the intracellular mechanism of the effects of tianeptine on M1 microglial cells. Recently, studies demonstrated an emerging role of the NLRP3 inflammasome platform, composed of NLRP3, ASC, and procaspase-1, in the inflammatory response. The activation of the NLRP3 inflammasome is tightly regulated, and it requires two independent signals. This is because basal protein expression of NLRP3 is very low; therefore, a priming step is required to act as “signal 1” to start its transcription [56]. This priming may be facilitated through the activation of Toll-like or NOD-like receptors. TLR4 acts as a major LPS signaling receptor in microglial cells, leading to the inflammatory response. The formation of an LPS-TLR4/myeloid differentiation protein 2 (MD-2) complex, and subsequent recruitment of an intracellular adaptor protein, MyD88, leads to the activation of mitogen-activated protein kinases (MAPKs) [57]. Among them, p-38 and ERK1/2 appear to be particularly involved in the production of pro-inflammatory mediators in microglial cells. In the presented study, we found that the pretreatment of microglia with tianeptine attenuated the LPS-evoked increase in TLR4 levels, and significantly reduced ERK1/2 phosphorylation. Since evidence indicated that LPS potentiates the phosphorylation of ERK1/2 and p38 in a dose- and time-dependent manner, leading to TNF-α release [58,59], the observed suppression of the ERK1/2 pathway may be engaged in the inhibition of the release of pro-inflammatory factors by tianeptine in LPS-stimulated microglia cells.

Nonetheless, our data also showed a suppressive impact of tianeptine on the activation of some subunits of the transcription factor NF-κB complex: p50, p65, and IκB. After its activation, IκB phosphorylation and degradation exposes the nuclear localization signals on the p50/p65 complex, leading to their nuclear translocation and the transcription of inflammasome-related components, including inactive NLRP3, proIL-1β, and proIL-18 [60,61]. We observed that pretreatment with tianeptine has a tendency to inhibit LPS-evoked IκB degradation in microglia. Additionally, our study showed that tianeptine suppressed the LPS-induced phosphorylation of a specific serine that is important in initiating transcription of the p65 NF-κB subunit in microglial cells [62]. Interestingly, recent findings postulated a role of caspase-3-dependent protein kinase Cδ (PKCδ) cleavage in the regulation of the NF-κB pathway in microglial cells [63]. Caspase-3 activation was found to occur in two stages that exert different roles. The cytoplasmic caspase-3 p19/p12 complex is responsible for the pro-inflammatory activation of microglia, whereas the nuclear caspase-3 p17/p12 complex promotes apoptosis in microglia [64]. We revealed that tianeptine inhibited the LPS-stimulated formation of the p19 caspase-3 subunit in microglia cells, as well as diminished the increase in PKCδ cleavage by the p19 caspase-3 subunit in LPS-treated cells (data not shown). Therefore, NF-κB modulation by tianeptine may be postulated to be complex and not clearly defined.

It should be noted that, once primed, the subsequent activation of NLRP3, referred to as “signal 2”, results in the oligomerization of NLRP3, and in the subsequent assembly of NLRP3, ASC, and procaspase-1 into a complex. This assembly triggers the transformation of procaspase-1 to caspase-1, as well as the production and secretion of mature IL-1β and IL-18 [61,65]. Importantly, in the presented study, we demonstrated, for the first time, that tianeptine was able to diminish the expression of NLRP3 and caspase-1 that was upregulated by LPS treatment. So far, three different models of NLRP3 activation were suggested, and one of them involves ROS formation, as many NLPR3 agonists were shown to promote ROS formation [66]. Recently, the production of ROS was stated to likely activate the inflammasome via intermediate pathways. Some data demonstrated that an increase in ROS leads to activation of the thioredoxin-interacting protein, which then binds to NLRP3, resulting in inflammasome activation [65]. Taking into account our presented results showing that tianeptine decreased LPS-induced ROS formation, whether this effect supports the suppressive action of this antidepressant on NLRP3 activation may necessitate further consideration. On the other hand, a recent study suggested that ROS are only necessary for priming the inflammasome [67]; therefore, further detailed studies of these mechanisms are required.

**Figure 8:**
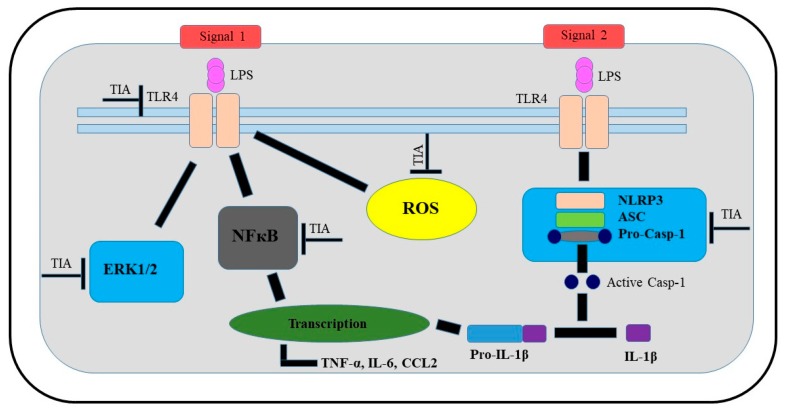
Schematic illustration of potential targets of beneficial tianeptine action in LPS-stimulated microglial cells. Abbreviations: TIA—tianeptine; LPS—lipopolysaccharide; TLR4—Toll-like receptor 4; NLRP3—the NOD-like receptor pyrin-containing 3 inflammasome; ASC—apoptosis-associated speck-like protein containing a caspase recruitment domain; ERK 1/2, extracellular signal-regulated kinases; NF-κB—nuclear factor; IL—interleukin; casp—caspase; ROS—reactive oxygen species; TNF—tumor necrosis factor; CCL2—chemokine.

In summary, the presented findings demonstrated that tianeptine exhibits protective and anti-inflammatory properties in LPS-stimulated microglia, confirmed by the ability of tianeptine to attenuate the LPS-evoked changes in microglia viability, and to reduce the levels of pro-inflammatory factors, including cytokines, chemokines, and nitric oxide, as well as reactive oxygen species production. However, we did not observe effects of tianeptine on the release of anti-inflammatory factors, which may suggest that tianeptine preferentially affects M1 microglia polarization. Furthermore, we demonstrated that the beneficial effects of tianeptine are primarily mediated through NLRP3 inflammasome-related pathways, involving TLR4, ERK1/2, NF-κB, and NLRP3, as well as caspase-1 inhibition (Figure 8). A better understanding of the multifaceted mechanisms of the actions of these atypical drugs on NLRP3 inflammasome signaling can be postulated to contribute to improvements in the efficiency of the pharmacotherapy of depression, particularly in that associated with microglia immunoactivation.

## 4. Materials and Methods

### 4.1. Animals

Sprague/Dawley rats (weighing 200–250 g upon arrival) obtained from Charles River (Sulzfeld, Germany) were kept under standard conditions (at a room temperature of 23 °C and a 12/12-h light/dark cycle with the light on at 8:00 a.m.) with food and water available ad libitum. Two weeks after arrival, vaginal smears were taken daily from female rats to determine the estrus cycle phase. On the proestrus day, females were placed with males for 12 h, and afterward, the presence of sperm in the vaginal smears was checked. Pregnant females were left undisturbed in their home cages. All experiments were carried out according to the National Institutes of Health Guide for the Care and Use of Laboratory Animals and were approved by the Local Ethics Committee, Kraków, Poland. Laboratory Animals Consent procedure (approval NO.: 1037/2013, 16 May 2013).

### 4.2. Cell Culture

Primary cultures of microglial cells were prepared from cortices of 1–2-day-old Sprague/Dawley rat pups as previously described [17]. Briefly, after decapitation, brains were removed, and cerebral cortices were cut into small pieces. Next, the minced tissue was incubated in Hank’s balanced salt solution (HBSS) dissecting medium (Gibco, Grand Island, NY, USA) with glucose, bovine serum albumin (BSA) and HEPES, as well as 0.025% trypsin for 20 min at 37 °C. The trypsin inhibitor from *Glycine max* (soybean) (Sigma-Aldrich, St. Louis, MO, USA) stopped the trypsinization process. A completely dissociated suspension of the tissue was prepared by mild trituration with a fire-polished Pasteur pipette. Then, cells were plated at a density of 3 × 10^5^ cells/cm^2^ in culture medium consisting of Dulbecco’s modified Eagle medium (DMEM) with GlutaMax and high glucose (4.5 g/L), supplemented with heat-inactivated 10% fetal bovine serum (FBS), 100 U/mL penicillin, and 0.1 mg/mL streptomycin on poly-l-lysine-coated 75-cm^2^ culture flasks. Three days later, the culture medium was removed and replaced with fresh medium. On the ninth day in vitro (37 °C, 95% O_2_/5% CO_2_), flasks were agitated on a horizontal shaker. After centrifugation (150× *g* for 10 min), cells were resuspended in culture medium, and cell viability was determined with trypan blue exclusion. The cells were plated at a final density of 1.2 × 10^6^ cells/well in six-well plates, 2 × 10^5^ cells/well in 24-well plates, or 4 × 10^4^ cells/well in 96-well plates. The purity of microglial cell cultures was assessed with an anti-Iba-1 antibody (sc-32725, Santa Cruz Biotechnology Inc., Dallas, TX, USA) and anti-CD11b antibody (201809, Biolegend, San Diego, CA, USA); more than 95% of cells were stained positively. The cells were used for experiments two days after plating.

### 4.3. Cell Treatment

In all presented experiments, cells were pretreated for 30 min with various concentrations of tianeptine (0.1, 1.0, 10.0, or 50 μM; Sigma-Aldrich, St. Louis, MO, USA), and were then stimulated with lipopolysaccharide (LPS; 100 ng/mL; *Escherichia coli* 0111:B4, Sigma-Aldrich, St. Louis, MO, USA). Control (unstimulated) cells were treated with vehicle (phosphate-buffered saline (PBS) buffer).

### 4.4. Cell Viability Test

Cell viability was determined using the tetrazolium salt 3-(4,5-dimethylthiazol-2-yl)-2,5-diphenyltetrazolium bromide (MTT; Sigma-Aldrich, St. Louis, MO, USA) assay. Microglial cells were seeded into 96-well plates at a density of 4 × 10^4^ cells per well with 100 μL of culture medium, and incubated for 48 h to allow cell adherence. At 24 h after pretreatment with different concentrations of tianeptine (0.1, 1.0, 10, and 50 μM), and then LPS (100 ng/mL) stimulation, MTT (at 0.15 mg/mL) was added to each well, which was subsequently incubated for 2 h at 37 °C. The culture medium was discarded, and 0.1 M HCl in isopropanol was added to dissolve the formazan dye. The obtained data were normalized to the absorbance value, measured using a multi-well spectrophotometer Infinite^®^ 200 PRO Detector (TECAN, Männedorf, Switzerland) at 570 nm in the vehicle-treated cells (100%), and are expressed as a percentage of the control ± standard error of the mean (SEM).

### 4.5. Flow Cytometry Analysis

To confirm the data obtained from the biochemical cell viability assay (MTT) we stained the microglial cells pretreated with tianeptine (1; 10 μM) for 30 min, and then with lipopolysaccharide (LPS; 100 ng/mL) for 24 h, with propidium iodide (PI). PI does not cross the cell membrane, but stains the DNA released from cells whose cell membrane was disintegrated. The microglial cells were incubated with the PI solution (10 μg/mL in PBS) for 10 min at 37 °C, followed by two centrifugations (1300 rpm, 5 min at room temperature) and washes in PBS. Approximately 1 × 10^4^ cells were analyzed using the BD FACSCanto II System and the BD FACSDiva*™* v5.0.1 software (BD Biosciences, Franklin Lakes, NJ, USA) in the fluorescence channel for PE (fluorescence channel for PerCP-Cy5-5-A (peridinin-chlorophyll proteins and far red fluorescence)). The PI-negative cells were considered to be normal, and the PI-positive cells were considered to be necrotic. The data are presented as a percentage of live cells ± SEM.

### 4.6. Nitric Oxide Release Assay

The Griess reaction was used to measure the NO secreted in microglial culture medium. After being pretreated with tianeptine for 30 min, and then with LPS (100 ng/mL) for 24 h, 50 μL of supernatant was collected and mixed with an equal volume of Griess reagent (0.1% *N*-1-naphthylethylenediamine dihydrochloride and 1% sulfanilamide in 5% phosphoric acid) in a 96-well plate, followed by incubation for 10 min at room temperature. Then, the absorbance was measured at 540 nm in a microplate reader (Infinite^®^ 200 PRO Detector, TECAN, Männedorf, Switzerland). The data were normalized to the NO released from vehicle-treated cells (100%), and are expressed as a percentage of the control ± SEM.

### 4.7. Measurement of Intracellular ROS Formation

To determine the intracellular level of ROS, we used the 2′,7′-dichlorofluorescin acetate (DCFH-DA) test according to the manufacturer’s instructions (Cell Biolabs, San Diego, CA, USA). After being pretreated with tianeptine for 30 min, and then with LPS (100 ng/mL) for 24 h, the cells were washed with PBS, and were then incubated with DCFH-DA (10 μM) for 30 min at 37 °C. DCFH-DA diffuses into cells and is deacetylated by cellular esterases to form non-fluorescent 2′,7′-dichlorodihydrofluorescin (DCFH), which is rapidly oxidized to the highly fluorescent 2′,7′-dichlorodihydrofluorescein (DCF) by ROS. The fluorescence intensity is proportional to the ROS levels within the cell cytosol. To detect the fluorescent intensity, we used an Infinite^®^ M1000 PRO microplate reader (TECAN, Männedorf, Switzerland) with excitation and emission wavelengths of 485 nm and 535 nm, respectively. The data were normalized to the level of ROS in vehicle-treated cells (100%), and are presented as the mean ± S.E.M.

### 4.8. Quantitative Real-Time Polymerase Chain Reaction (qRT-PCR)

Total RNA was extracted from microglial cells using the RNA Isolation Kit (RNeasy Mini Kit, Applied, Biosystems, Foster City, CA, USA) following the manufacturer’s instructions. The RNA concentration was determined using a NanoDrop Spectrophotometer (ND/1000 UV/Vis; Thermo Fisher NanoDrop, Waltham, MA, USA). Identical amounts of RNA (1 μg) were reverse transcribed into complementary DNA (cDNA) using a commercial RT-PCR kit (High-Capacity cDNA Reverse Transcription Kit, Applied Biosystems, Foster City, CA, USA) according to the manufacturer’s instructions. The cDNA was subsequently amplified using TaqMan probes and primers for the following genes: IL-1β (Rn00580432_m1), IL-6 (Rn01410330_m1), IL-18 (Rn01422083_m1), IL-4 (Rn01456866_m1), IL-10 (Rn01644839_m1), TGF-β (Rn01418715_m1), NLRP3 (Rn04244620_m1), ASC (Rn00597229_g1), Casp-1 (Rn00562724_m1), IGF-1 (Rn00710306_m1), TNF-α (Rn00562055_m1), chemokine CC motif ligand 2 (CCL2; Rn00580555_m1), CD40 (Rn01423590_m1), and MHC II (Rn01424725_m1), obtained from Life Technologies, Carlsbad, CA, USA, with the FastStart Universal Probe Master (Rox) kit (Roche, Basel, Switzerland). Next, the amplification was carried out in a total volume of 20 μL containing 1× FastStart Universal Probe Master (Rox) mix, cDNA used for the PCR template, TaqMan forward and reverse primers, and 250 nM of hydrolysis probe labeled with the fluorescent reporter dye, fluorescein (FAM), at the 5′-end, and a quenching dye at the 3′-end. Thermal cycling conditions were as follows: 2 min at 50 °C and 10 min at 95 °C, followed by 40 cycles at 95 °C for 15 s and at 60 °C for 1 min. The threshold value (*C*_t_) for each sample was set in the exponential phase of PCR, and the ΔΔ*C*_t_ method was used for data analysis. Hypoxanthine guanine phosphoribosyl transferase (HGPRT; Rn01527840_m1) was used as the reference gene.

### 4.9. Western Blotting Analysis

The western blot analyses were conducted as described previously [68,69]. Concisely, the cells were lysed with a radioimmunoprecipitation assay (RIPA) lysis buffer (Sigma-Aldrich, St. Louis, MO, USA) with a protease inhibitor cocktail (Sigma-Aldrich), phosphatase inhibitor cocktail (Sigma-Aldrich), 1 mM sodium orthovanadate (Sigma-Aldrich), and 1 mM phenylmethanesulfonyl fluoride (Sigma-Aldrich). The lysates (equal amounts of protein) and the buffer (4 × Laemmi buffer, Roche, Basel, Switzerland) were then mixed and boiled for 6 min before they were loaded onto the gel. Proteins were separated using 4–20% Criterion^TM^ TGX^TM^ Precast Midi Protein Gel, 26 well (Bio-Rad, Hercules, CA, USA) under constant voltage (200 V), and were then transferred electrophoretically to polyvinylidene fluoride (PVDF) membranes (Trans-Blot Turbo; Bio-Rad). Next, the membranes were washed twice with Tris-buffered saline (TBS), pH = 7.5, blocked in 5% bovine serum albumin for 1 h at room temperature, and incubated overnight at 4 °C with the following primary antibodies that were diluted in a SignalBoost Immunoreaction Enhancer Kit (Millipore, Warsaw, Poland): anti-p-65 (sc-372), anti-phospho-p-65 (sc-33039), anti-inhibitor of κB kinase (IκB; sc-1643), anti-p-38 (sc-7149), anti-phospho-p38 (sc-101759), anti-ERK1/2 (sc-135900), anti-phospho-ERK1/2 (sc-16982), anti-JNK (sc-7345), anti-phospho-JNK (sc-12882); all antibodies were from Santa Cruz Biotechnology, Inc., USA. After the blots were washed with TBS containing 0.1% Tween-20 (TBST) four times, they were incubated with a horseradish peroxidase-linked secondary antibody (anti-rabbit/anti-mouse/anti-goat immunoglobulin G (IgG); Santa Cruz Biotechnology, Inc., Dallas, TX, USA) at room temperature for 1 h. Afterward, the membranes were washed four times with large volumes of TBST. The immune complexes were detected using the Pierce^®^ ECL Western Blotting Substrate (Thermo Fisher, Pierce Biotechnology, Carlsbad, CA, USA), and were visualized using a Fujifilm LAS-1000 System (Fuji Film, Tokyo, Japan). The blots were washed two times for 5 min each in TBS; stripped using stripping buffer containing 100 μL of Tris-HCL (pH = 6.7), 2% SDS, and 700 μL of 2-mercaptoethanol (all from Sigma Aldrich, St. Louis, MO, USA); washed two additional times for 5 min each in TBS; blocked; and reprobed with an antibody against β-actin (MAB374, Millipore, Warsaw, Poland) as an internal loading control at a dilution of 1:15,000 in a SignalBoost Immunoreaction Enhancer Kit. In our experiment, most of the membranes were stripped twice. The relative levels of immunoreactivity were densitometrically quantified using the Fujifilm Multi Gauge software (Fuji Film, Tokyo, Japan).

### 4.10. Enzyme-Linked Immunosorbent Assay (ELISA) 

The various concentrations of tianeptine (Sigma-Aldrich) were added to the primary microglia (2 × 10^5^ cells/well in 24-well plate) for 30 min, and then, the cells were stimulated with LPS (100 ng/mL). The microglial cell medium was collected at 24 h after treatment to assess TNF-α, IL-1β, IL-6, IL-18, IL-4, IL-10, TGF-β, and CCL2 levels. The protein levels of the cytokines, TNF-α, IL-1β, (Rat TNF-alpha Quantikine Elisa Kit, Rat IL-1beta/IL-1F2 Quantikine Elisa kit; both R&D Systems, Minneapolis, MN, USA), IL-6, IL-4, IL-10, TGF-β, CCL2 (Elisa kit for interleukin 6 (IL-6), Elisa kit for Interleukin 4 (IL-4), Elisa kit for Interleukin 10 (IL-10), Elisa kit for Transforming Growth Factor Beta (TGF-β), Elisa kit for monocyte chemotactic protein 1 (CCL2/MCP-1); all USCN Life Science Inc., Wuhan, China), and IL-18 (IL-18 Elisa kit, Invitrogen, Carlsbad, CA, USA), in the culture medium were measured using commercially available enzyme-linked immunosorbent assay kits according to the manufacturers’ instructions. Additionally, 24 h after treatment, the cells were lysed with an RIPA lysis buffer (Sigma-Aldrich) with a protease inhibitor cocktail (Sigma-Aldrich), phosphatase inhibitor cocktail (Sigma-Aldrich), 1 mM sodium orthovanadate (Sigma-Aldrich), and 1 mM phenylmethanesulfonyl fluoride (Sigma-Aldrich). The protein levels of NLRP3, ASC (Elisa kit for Pyrin Domain-Containing Protein 3, Elisa kit for PYD and CARD Domain-Containing Protein; both USCN Life Science Inc., Wuhan, China), Casp-1 (caspase-1 Elisa kit; EIAab Wuhan Science, Wuhan, China), IGF-1 (Rat IGF-1 Elisa kit, R&D Systems, Minneapolis, MN, USA), iNOS (Rat Inducible Nitric Oxide Synthase Elisa kit, Cusabio, Houston, TX, USA), and TLR4 (Rat Toll-like receptor 4 Elisa kit, Cusabio, Houston, TX, USA) in the microglial lysates were measured using commercially available enzyme-linked immunosorbent assay kits according to the manufacturers’ instructions. The detection limits were as follows: TNF-α, 5 pg/mL; IL-1β, 5 pg/mL; IL-6, 6.2 pg/mL; IL-18, 4 pg/mL; IL-4, 2.85 pg/mL; IL-10, 3.2 pg/mL; TGF-β, 5.5 pg/mL; CCL2, 0.055 ng/mL; NLRP3, 0.123 ng/mL; ASC, 0.065 ng/mL; Casp-1, 78 pg/mL; IGF-1, 3.5 pg/mL; iNOS, 0.195 IU/mL; and TLR4, 0.156 ng/mL. Inter-assay precision values were as follows: TNF-α, <8.8%; IL-1β, <4.4%; IL-6, <12%; IL-18, <4.5%; IL-4, <12%; IL-10, <12%; TGF-β, <12%; CCL2, <12%; NLRP3, <12%; ASC, <12%; Casp-1, <7,8%; IGF-1, <12%; iNOS, <10%; and TLR4, <10%. Intra-assay precision values were as follows: TNF-α, <2.1%; IL-1β, <3.9%; IL-6, <10%; IL-18, <3.5%; IL-4, <10%; IL-10, <10%; TGF-β, <10%; CCL2, <10%; NLRP3, <10%; ASC, <10%; Casp-1, <5,3%; IGF-1, <10%; iNOS, <8%; and TLR4, <8%.

### 4.11. Statistical Analysis

Statistical analysis was performed using the Statistica 10.0 Software (StatSoft, Palo Alto, CA, USA). The results presented in the paper were derived from three independent primary microglial cultures, while “n” for each culture was 2–5. All data are presented as the mean ± SEM (standard error of the mean). The results of the cell viability and death processes are presented as the mean ± SEM percentage of control (vehicle-treated cells). The data obtained in the ELISA study are presented as the mean ± SEM percentage of control (vehicle-treated cells); those for RT-PCR are presented as an average fold ± SEM, and for western blot analysis, the results are presented as the mean ± SEM percentage of control (vehicle-treated cells). The normality of the variable distribution and the homogeneity of the variances were checked by the Shapiro–Wilk test and Levene’s test, respectively. The results were statistically evaluated using two-way analysis of variance (ANOVA), followed, when appropriate, by Duncan post-hoc tests to assess the differences between the treatment groups. A *p*-value less than or equal to 0.05 was considered as statistically significant. Significant differences from the control group (vehicle-treated cells) are indicated by * *p* < 0.05; differences between LPS-treated groups and pre-treated with tianeptine and then stimulated with LPS groups are indicated by ^#^ *p* < 0.05. All graphs were prepared using GraphPad Prism 5.

## Figures and Tables

**Table 1:** The effect of tianeptine (TIA) on microglia activation markers (major histocompatibility complex class II (MHC II) and cluster of differentiation 40 (CD40)), pro-inflammatory factors (interleukin (IL)-1β, IL-18, tumor necrosis factor alpha (TNF-α), IL-6, and chemokine CC motif ligand 2 (CCL2)) and anti-inflammatory factors (IL-4, IL-10, transforming growth factor (TGF-β), and insulin-like growth factor 1 (IGF-1)), measured using qRT-PCR in vehicle- and lipopolysaccharide (LPS)-treated primary microglial cells. Microglial cells were pretreated with tianeptine (TIA; 10 μM) for 30 min, and then with lipopolysaccharide (LPS; 100 ng/mL). The data are presented as an average fold ± standard error of the mean (SEM) from independent experiments. The results were statistically evaluated using two-way analysis of variance (ANOVA) with a Duncan post-hoc test to assess the differences between the treatment groups. Significant differences from the control group (vehicle-treated cells) are indicated by * *p* < 0.05; differences between LPS-treated groups and pre-treated with tianeptine and then stimulated with LPS groups are indicated by ^#^ *p* < 0.05. The bold data shows statistically significant results.

Factor	Gene Expression			
	Control	LPS	Control + TIA10	LPS + TIA10
MHCII	1.00 ± 0.06	2.38 ± 0.29 *	1.00 ± 0.03	**1.30 ± 0.07 ^#^**
CD40	1.00 ± 0.06	2.34 ± 0.15 *	1.02 ± 0.04	**1.46 ± 0.05 ^#^**
IL-1β	1.00 ± 0.04	3.34 ± 0.32 *	1.03 ± 0.03	**1.48 ± 0.23 ^#^**
IL-18	1.00 ± 0.07	3.06 ± 0.27 *	0.96 ± 0.08	**1.39 ± 0.07 ^#^**
TNF-α	1.00 ± 0.04	4.50 ± 0.43 *	0.94 ± 0.05	**2.06 ± 0.32 ^#^**
IL-6	1.00 ± 0.03	1.51 ± 0.10 *	0.97 ± 0.03	**1.26 ± 0.04 ^#^**
CCL2	1.00 ± 0.09	2.69 ± 0.31 *	1.06 ± 0.09	**1.37 ± 0.12 ^#^**
IL-4	1.00 ± 0.04	0.73 ± 0.05	1.78 ± 0.82	1.01 ± 0.16
IL-10	1.01 ± 0.06	8.68 ± 1.31 *	0.69 ± 0.15	9.74 ± 1.49 *
TGF-β	1.00 ± 0.03	0.37 ± 0.05 *	0.58 ± 0.05 *	0.58 ± 0.01 *
IGF-1	1.06 ± 0.16	0.0081 ± 0.0022 *	0.67 ± 0.19	0.0242 ± 0.0032 *

## Supplementary Materials

Supplementary materials can be found at http://www.mdpi.com/1422-0067/19/7/1965/s1.

## Abbreviations

AP-1	activator protein-1
ASC	apoptosis-associated speck-like protein containing a caspase recruitment domain
Casp-1	caspase-1
CD40	cluster of differentiation 40
CNS	central nervous system
CX3CL1	fractalkine
CX3CR1	fractalkine receptor
DCFH	2′,7′-dichlorodihydrofluorescin
DCFH-DA	2′,7′-dichlorofluorescin diacetate
DMEM	Dulbecco’s modified Eagle medium
ERK 1/2	extracellular signal–regulated kinases
FBS	fetal bovine serum
HBSS	Hank’s balanced salt solution
HPA	hypothalamus-pituitary-adrenal
IκB	inhibitor of κB kinases
IGF-1	insulin-like growth factor
IL	interleukin
iNOS	inducible nitric oxide synthase
JNK	c-Jun N-terminal kinases
CCL2/MCP-1	chemokine CC motif ligand 2/monocyte chemoattractant protein-1
MD-2	myeloid differentiation protein-2
MHC II	major histocompatibility complex class II
MTT	tetrazolium salt 3-(4,5-dimethylthiazol-2-yl)-2,5-diphenyltetrazolium bromide
NF-κB	nuclear factor kappa-light-chain-enhancer of activated B cells
NLRP3	NOD-like receptor pyrin-containing 3 inflammasome
NO	nitric oxide
PBS	phosphate-buffered saline
PKCδ	protein kinase C δ type
RIPA	radioimmunoprecipitation assay
ROS	reactive oxygen species
SSRIs	selective serotonin uptake inhibitors
TBS	Tris-buffered saline
TCAs	tricyclic antidepressants
TGF-β	transforming growth factor β
TLR	Toll-like receptor
TNF-α	tumor necrosis factor α
