# Supplementary material for: Targeting the NLRP3 Inflammasome-Related Pathways via Tianeptine Treatment-Suppressed Microglia Polarization to the M1 Phenotype in Lipopolysaccharide-Stimulated Cultures
Source: Int J Mol Sci. 2018 Jul 5;19(7):1965. doi: 10.3390/ijms19071965 (PMC6073715; doi:10.3390/ijms19071965)
Supplement: Supplementary file 1 [file ijms-19-01965-s001.ppt]

## Slide 1
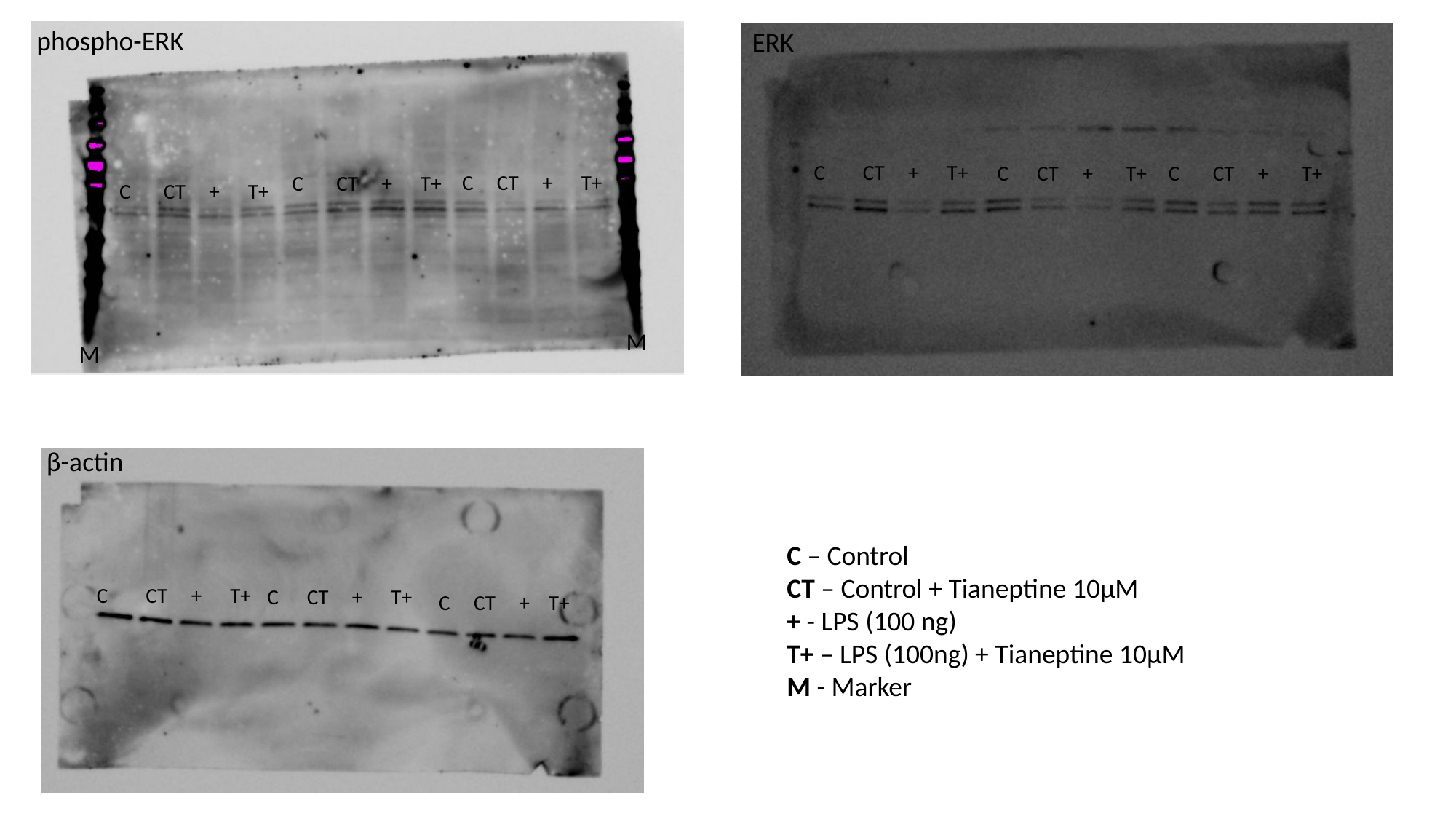

phospho-ERK
ERK
C CT + T+
C CT + T+
C CT + T+
C CT + T+
 C CT + T+
C CT + T+
M
M
β-actin
C – Control
CT – Control + Tianeptine 10µM
+ - LPS (100 ng)
T+ – LPS (100ng) + Tianeptine 10µM
M - Marker
C CT + T+
C CT + T+
C CT + T+

## Slide 2
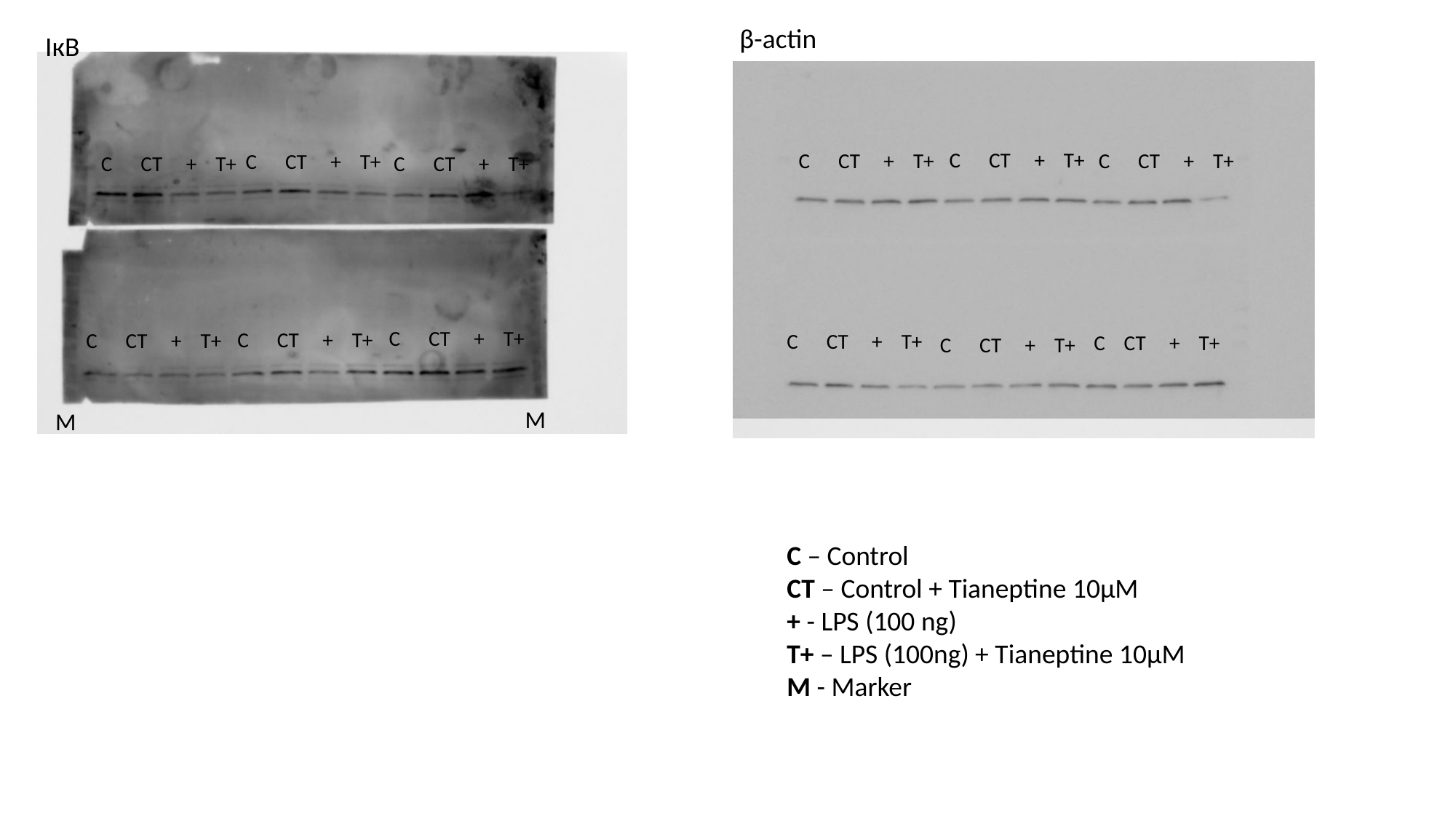

β-actin
IкB
C CT + T+
C CT + T+
C CT + T+
C CT + T+
C CT + T+
C CT + T+
C CT + T+
C CT + T+
C CT + T+
C CT + T+
C CT + T+
C CT + T+
M
M
C – Control
CT – Control + Tianeptine 10µM
+ - LPS (100 ng)
T+ – LPS (100ng) + Tianeptine 10µM
M - Marker

## Slide 3
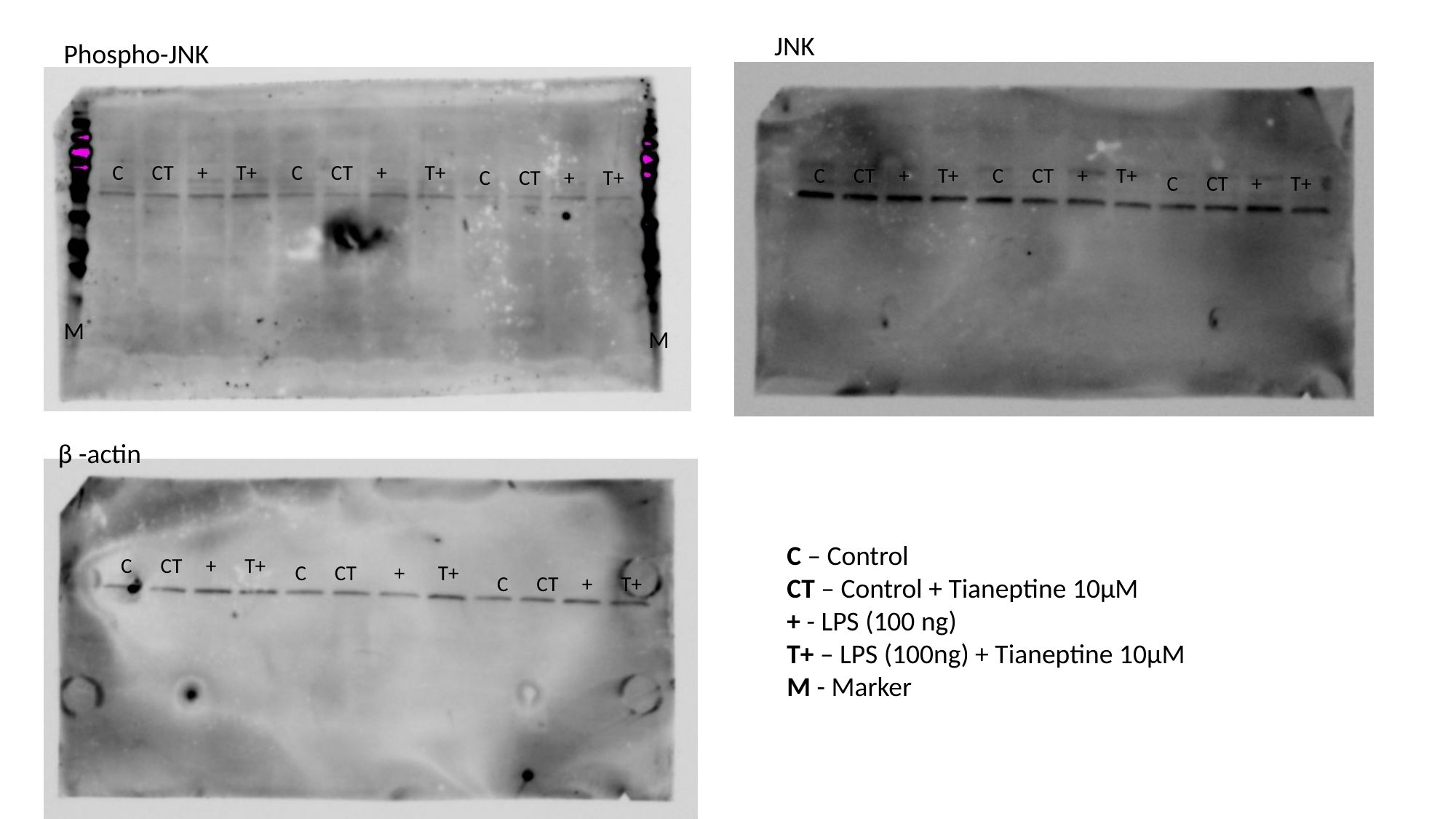

JNK
Phospho-JNK
C CT + T+
C CT + T+
C CT + T+
C CT + T+
C CT + T+
C CT + T+
M
M
β -actin
C – Control
CT – Control + Tianeptine 10µM
+ - LPS (100 ng)
T+ – LPS (100ng) + Tianeptine 10µM
M - Marker
C CT + T+
C CT + T+
C CT + T+

## Slide 4
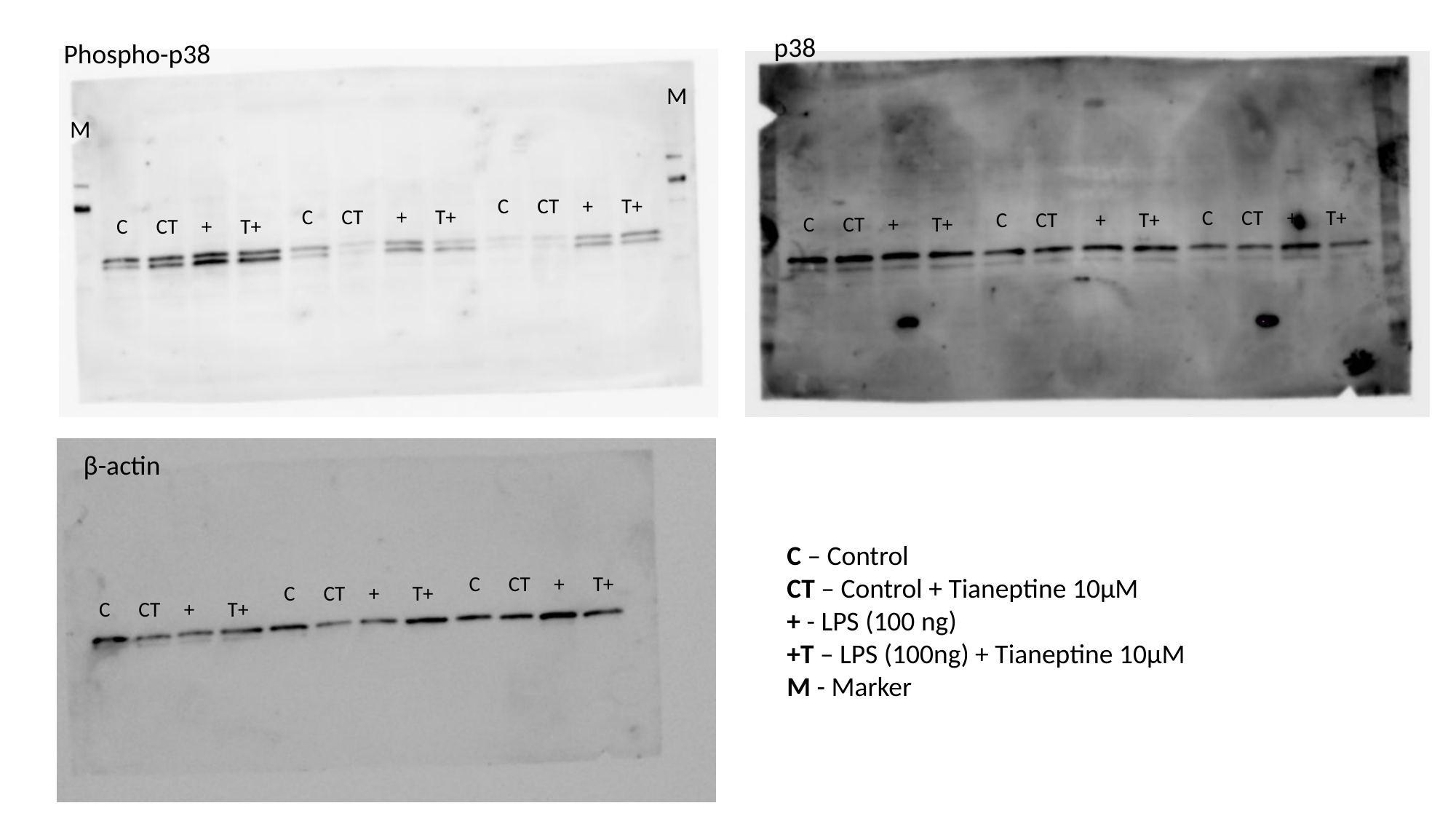

p38
Phospho-p38
M
M
C CT + T+
C CT + T+
C CT + T+
C CT + T+
C CT + T+
C CT + T+
β-actin
C – Control
CT – Control + Tianeptine 10µM
+ - LPS (100 ng)
+T – LPS (100ng) + Tianeptine 10µM
M - Marker
C CT + T+
C CT + T+
C CT + T+

## Slide 5
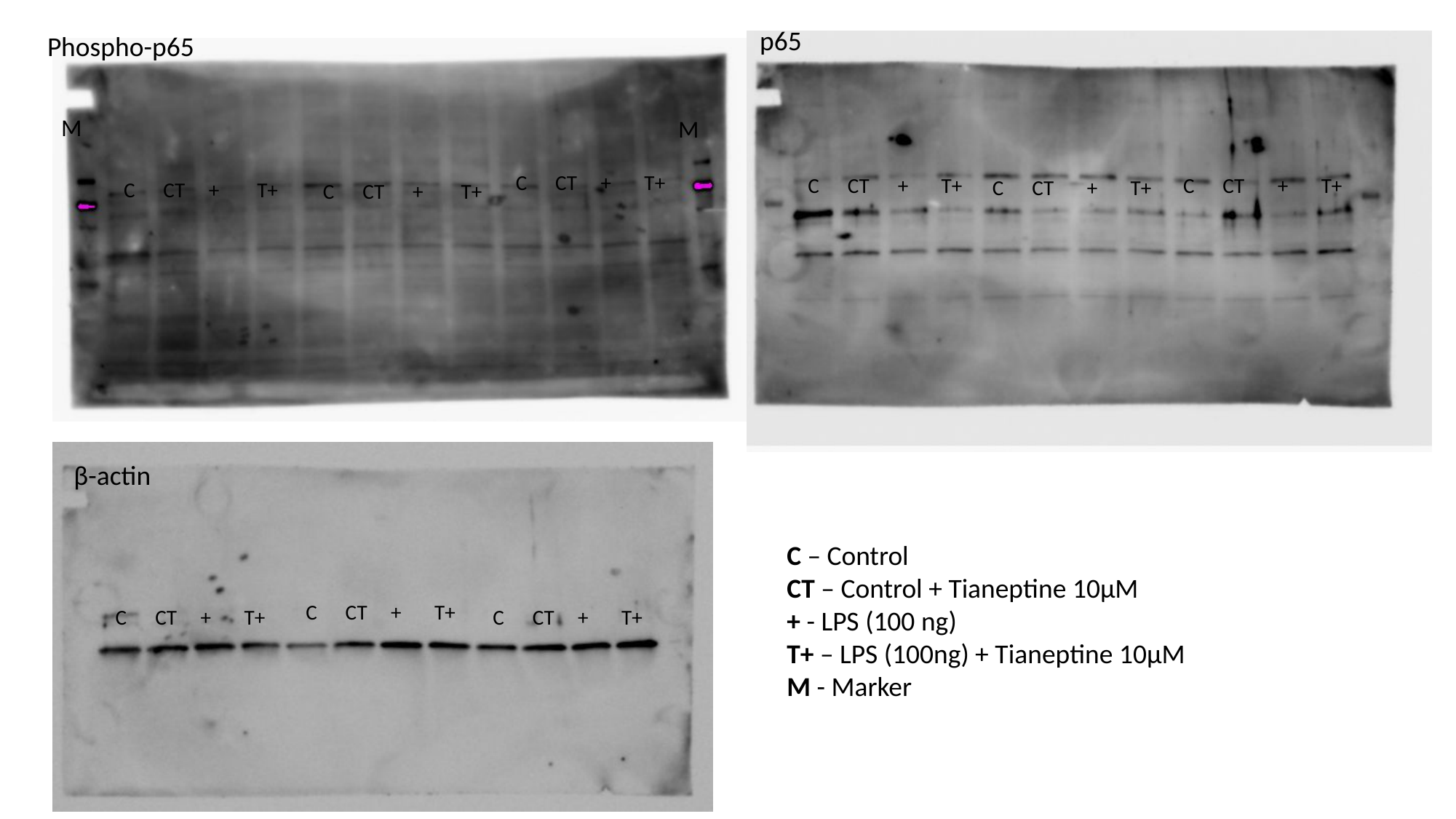

p65
Phospho-p65
M
M
C CT + T+
C CT + T+
C CT + T+
C CT + T+
C CT + T+
C CT + T+
β-actin
C – Control
CT – Control + Tianeptine 10µM
+ - LPS (100 ng)
T+ – LPS (100ng) + Tianeptine 10µM
M - Marker
C CT + T+
C CT + T+
C CT + T+
